# Phytochemicals as Modulators of Long Non-Coding RNAs and Inhibitors of Cancer-Related Carbonic Anhydrases

**DOI:** 10.3390/ijms20122939

**Published:** 2019-06-15

**Authors:** Tayebeh Saghafi, Ramezan Ali Taheri, Seppo Parkkila, Reza Zolfaghari Emameh

**Affiliations:** 1 Department of Energy and Environmental Biotechnology, National Institute of Genetic Engineering and Biotechnology (NIGEB), Tehran, P.O. Box 14965/161, Iran; tb.sghfi@gmail.com; 2 Nanobiotechnology Research Center, Baqiyatallah University of Medical Sciences, Tehran, P.O. Box 14965/161, Iran; taheri@bmsu.ac.ir; 3 Faculty of Medicine and Health Technology, Tampere University, FI-33520 Tampere, Finland; seppo.parkkila@tuni.fi; 4 Fimlab Laboratories Ltd. and Tampere University Hospital, FI-33520 Tampere, Finland

**Keywords:** cancer, phytochemicals, long non-coding RNA (lncRNA), modulator, carbonic anhydrase

## Abstract

Long non-coding RNAs (lncRNAs) are classified as a group of transcripts which regulate various biological processes, such as RNA processing, epigenetic control, and signaling pathways. According to recent studies, lncRNAs are dysregulated in cancer and play an important role in cancer incidence and spreading. There is also an association between lncRNAs and the overexpression of some tumor-associated proteins, including carbonic anhydrases II, IX, and XII (CA II, CA IX, and CA XII). Therefore, not only CA inhibition, but also lncRNA modulation, could represent an attractive strategy for cancer prevention and therapy. Experimental studies have suggested that herbal compounds regulate the expression of many lncRNAs involved in cancer, such as HOTAIR (HOX transcript antisense RNA), H19, MALAT1 (metastasis-associated lung adenocarcinoma transcript 1), PCGEM1 (Prostate cancer gene expression marker 1), PVT1, etc. These plant-derived drugs or phytochemicals include resveratrol, curcumin, genistein, quercetin, epigallocatechin-3-galate, camptothcin, and 3,3′-diindolylmethane. More comprehensive information about lncRNA modulation via phytochemicals would be helpful for the administration of new herbal derivatives in cancer therapy. In this review, we describe the state-of-the-art and potential of phytochemicals as modulators of lncRNAs in different types of cancers.

## 1. Introduction

It is known that only about 2% of the human genome is transcribed into proteins or regulatory elements, while the rest of the genome is either non-coding or transcribed into RNA, with no possibility for translation to any protein, although it is biologically active [1]. These transcribed RNAs are called non-coding RNAs (ncRNAs) [2]. ncRNAs are classified into two groups: (1) small non-coding RNAs, which are about 22 nucleotides, and (2) long non-coding RNAs (lncRNAs), which are longer than 200 nucleotides with no open reading frame (ORF) restriction [3]. H19 was the first lncRNA reported in 1990 by Brannan et al. [4]. H19 is an imprinted oncofetal RNA, the expression of which decreases after birth, while the overexpression of H19 lncRNA has been reported in many cancer types in humans [5]. The latest lncRNAs have been reported in NONCODE [6], which is a comprehensive database covering non-coding RNAs. It presents data for 17 species, including 172,216 (as of February 2019) human lncRNA transcripts, which are able to regulate cell growth, development, differentiation, and gene expression [7]. Furthermore, lncRNAs play an important role in the occurrence of various diseases, such as cancer, whenever they are dysregulated [8]. They take part in cellular proliferation, apoptosis, and migration in a variety of cancers [9], such as breast cancer [10], prostate cancer [11], renal cancer [12], pancreatic cancer [13], and lung cancer [14]. Recently, numerous studies have introduced new types of drugs derived from plants (phytochemicals), which regulated the expression of several lncRNAs in cancer cells with no side effects [15].

It is well-documented that healthy nutrition prevents cancer. In contrast, the consumption of red meat [16] and high-fat diets [17] are associated with cancer induction. On the other hand, it was shown that vitamins B, D, and E [18,19,20] prevent different kind of tumors, including colorectal adenomas and prostate cancer. In addition, many other factors have been associated with cancer prevention or therapy as potential targets. They also include carbonic anhydrase (CA) enzymes, especially CA II, CA IX, and CA XII, which are overexpressed in certain cancers. Cas, as the major regulators of pH homeostasis, are induced by hypoxia and aid cancer cell survival [21,22]. Studies have shown the overexpression of cancer-related CAs, such as CA IX, in tumor cells, while their expression in normal cells is often low [23]. These facts and a number of recent publications suggest that cancer-related CAs are, indeed, potential and promising anti-cancer targets [22]. These CAs can be efficiently inhibited using various types of inhibitors, such as 7-aryl-triazolyl-substituted sulfocoumarins [24], acetazolamide [25,26,27,28,29,30,31,32], 6-ethoxy-2-benzothiazolesulfonamide (EZA) [33], benzene sulfonamides [34], 1,3,4-thiadiazole-2-sulfonamide [35], and sulfamide-related compounds [36]. CAs may also be modulated by lncRNAs via the administration of phytochemical compounds.

Phytochemicals are non-nutritive chemical components taken from various vegetables, fruits, beverages, and other green plants. Generally, the mechanism of action of these compounds occurs through the simulation of hormones, while they are known by their anti-oxidant and anti-inflammatory activities in cells [37,38,39,40]. To date, many phytochemicals have been identified and several are considered potential drugs due to their anticancer properties. They can be used as single chemopreventive drugs or synergistically with other routine anticancer drugs. This kind of anticancer drug administration can improve the efficacy of the treatment strategy, and optimally, with minimal or no side effects [41,42]. It has been suggested that phytochemicals act through the modulation of different signaling pathways via the regulation of significant molecular targets [43,44]. We hypothesize that they could also function by modulating the expression of enzymes, such as CAs, which are important for carcinogenic processes. In this review, we describe the state-of-the-art of how lncRNAs and cancer-related CAs could be modulated and inhibited by defined phytochemicals as an additional option for cancer prevention and treatment.

### 1.1. Biogenesis of lncRNA

**Figure 1:**
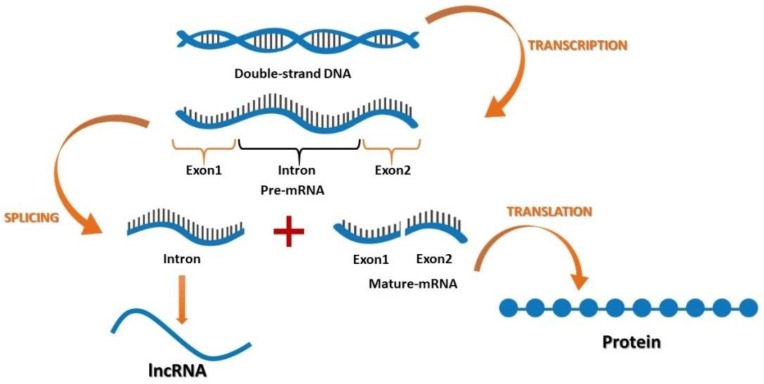
The flow of genetic information encoding for mRNA and long non-coding RNA (lncRNA).

After the discovery of coding and non-coding parts of the genome, it was suggested that non-coding sections may play an important role in cellular activities [45]. Furthermore, recent findings have suggested that lncRNAs function in various cancers, where their contribution is based on developmental and tissue specific expression patterns [46,47,48,49,50,51,52,53,54]. Both coding and non-coding genes carry genetic information with different functions. According to their location in the genome, lncRNAs can be divided into four groups: (1) the intergenic lncRNAs, which are located between two genes; (2) the sense or antisense lncRNAs, which may overlap with an exon of another transcript in the same or opposite direction; (3) the intronic lncRNAs, which reside within an intron and do not overlap with any exon; and (4) the processed transcripts, which reside in a locus where none of the transcript has an ORF and thus, do not fit into any other categories because of structural complexity (Figure 1).

Kapranov et al. [55] declared that lncRNAs can be generated during RNA purification, where some sections appeared in a silica column. The researchers suggested that this demonstrated a random cutoff from RNA with more than 200 nucleotides which can bind to the RNA purification column, while it is not functional. Then, they identified three major properties of this RNA molecule: (1) it might have or does not have an ORF for coding more than 100 amino acids; (2) there is no need for this section to produce a protein, but is still functional [56] and ; (3) it can contain both coding and non-coding domains [57,58,59].

The current and widely accepted description of lncRNAs states that they are RNA molecules which do not fit in any other classes of RNAs [60,61], do not contain any ORF restriction, and do not encode any protein. According to various studies, lncRNAs include newly discovered segments of RNA, which are overexpressed in different cancer cell types (Table 1). Cancer studies have indicated that HOTAIR, as one of the best known lncRNAs, is overexpressed in numerous malignancies, including breast, colorectal, renal, and pancreatic cancers [62]. On the other hand, it has been shown that downregulation of HOTAIR expression leads to activation of the PI3K-AKT-mTOR1 signaling pathway in most cancers [63,64]. Inhibition of HOTAIR also upregulated miR-454-3p expression in chondrosarcoma, which consequently repressed the STAT3 signaling pathway [65]. The overexpression of HOTAIR in renal cancer decreased apoptosis and increased cell migration and invasion [66]. Tang et al. demonstrated that the knockdown of HOTAIR released caspase-2 through activation of the apoptosis signaling pathway during cancer treatment [67].

Upregulation of MALAT1 was observed in many kinds of cancers, like oral, bladder, and colorectal carcinomas, as well as osteosarcoma [94,95,96,97]. The levels of H19 increased in various cancers, such as gastric and gallbladder carcinomas [89,90,91]. Other lncRNAs, such as PCGEM1, HOTAIR, and AK001796, were overexpressed in most cases of prostate cancer and phytochemical-induced anticancer activities [82,103,112,113]. Additionally, the role of HULC in promoting oral and liver cancers was proposed [75,76,114]. In pancreatic cancer, the expression of HOTAIR and PVT1 was upregulated [104,105,106]. Moreover, HOTAIR overexpression induced thyroid cancer in humans [83].

### 1.2. Modulation of lncRNA by phytochemicals

**Figure 2:**
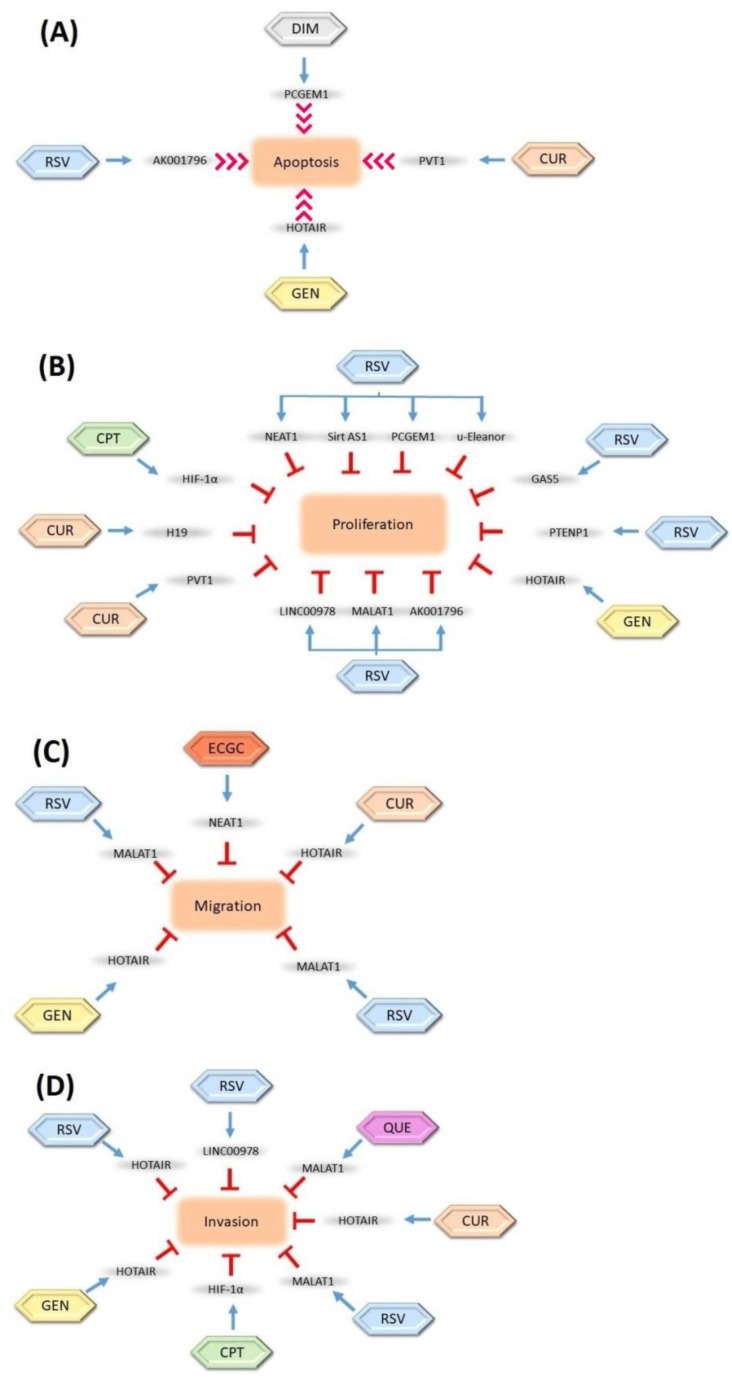
Regulation of long non-coding RNAs (lncRNAs) by natural compounds and their inhibition effects on cell (**A**) apoptosis, (**B**) proliferation, (**C**) migration, and (**D**) invasion. The inhibition relationships are denoted as red stop symbols, whereas positive interactions are denoted as normal blue arrows. CUR: Curcumin, GEN: Genistein, RSV: Resveratrol, ECGC: Epigallocatechin-3-gallate, CPT: Camptothecin, DIM: 3,3-diindolylmethane, QUE: Quercetin. The blue arrows show the modulation roles of phytochemicals, the red arrows show the induction role of phytochemicals, and the T bars show the inhibition role of phytochemicals on the lncRNAs.

lncRNAs are considered great targets for anticancer studies due to their potential tumor suppressor abilities. Several studies have suggested that the modulation of lncRNAs with various phytochemicals could be a novel option in cancer therapy. It has been clearly indicated that these lncRNAs are regulated by defined phytochemicals (Figure 2).

These phytochemicals include various compounds, some of which are presented in the following paragraphs.

### 1.3. Camptothecin (CPT)

Camptothecin (CPT, C20H16N2O4) is an alkaloid derived from a Chinese tree Camptotheca acuminate (happy tree). CPT has an inhibitory role in topoisomerase I and possesses antitumor activity [115,116,117]. CPT was demonstrated to suppress hypoxia-inducible factor 1 alpha (HIF-1α) -antisense RNA 1 in different human cancer types [77,106,115]. CPT also induces apoptosis in cardiovascular and kidney carcinomas, which results in an enhancement of the expression of antisense lncRNA. In another study, CPT treatment was shown to regulate the expression of lncRNA HIF-1α synergically with miR-17-5-p and miR-155 [78]. CPT has the ability to reduce CA IX expression in the cancer zone through the inhibition of angiogenesis and hypoxia. CPT has been conjugated to a linear, cyclodextrin-polyethylene glycol (CD-PEG) copolymer to form CRLX101 as a nanoparticle-drug conjugate (NDC). The conjugation step revealed that CRLX101 was more efficient than CPT in terms of the induction of apoptosis and supression of angiogenesis [118,119,120,121] (Table 2).

### 1.4. Curcumin

Curcumin (diferul[84]oylmethane) (C21H20O6 or C21H20O6) is a polyphenol derived from a perennial herbaceous plant, Curcuma longa [122]. This spicy yellow powder is used as an anti-inflammatory, antimicrobial, and antioxidant in traditional Asian medicine [123,124]. Curcumin acts as a chemopreventive and chemotherapeutic drug against various types of tumors, and is an important lncRNA regulator in cancers [125]. Petric et al. have shown that curcumin has an inhibitory effect on some oncogenic signaling pathways, including NF-kB, and induces apoptotic processes in breast cancer [38]. In another study, curcumin inhibited the overexpression of GAS5 in lung cancer by affecting signaling pathways, such as NF-kB, STAT3, and PI3K/Akt, to suppress tumor cell proliferation [126]. Curcumin also caused the modulation of tumor suppressor HOTAIR in pancreatic cancer [13], prostate cancer [88], hepatocellular carcinoma (HCC) [84,86], nasopharyngeal carcinoma (NPC) [81], breast cancer [87], lung cancer [80], and renal cancer [62,84,85,86,87,104,127]. It seems that the upregulation of HOTAIR has a controversial effect in terms of the occurrence of different cancer types and response to therapy methods, so radioresistance in breast cancer is enhanced by upregulated HOTAIR [87]. In addition, the expression level of HOTAIR is higher in renal cell carcinoma in comparison with normal kidney cells and a correlation has been shown between the upregulation of HOTAIR and distant metastasis in renal cell carcinoma malignancy [128]. Therefore, curcumin acts as a HOTAIR modulator, which consequently modulates the miR-19/PTEN/AKT/p53 axis in cancers [129].

Another tumor suppressor, H19, is induced by curcumin and directly inhibits p53 activation [90]. Overexpression of p53 can lead to colorectal and pancreatic cancer [130,131,132]. In nasopharyngeal carcinoma, high levels of expression have been demonstrated for six lncRNAs, including AF086415, AK095147, RP1-179N16.3, MUDENG, AK056098, and AK294004 [68,69]. Curcumin suppresses the expression of these lncRNAs and can tenderize cancer cells to radiotherapy [133]. On the other hand, different types of Cas, including isozymes I, II, IX, and XII, which are overexpressed in several cancers, are inhibited by curcumin and it’s phenolic compounds [134,135,136]. In addition, sulfonamides containing curcumin inhibited CA I [137]. The combination of curcumin with other factors can be a potent strategy in the treatment of tumor cells. This includes the combination of curcumin with glucose restriction [138] and dopamine-related compounds as phenolic sulfonamides and inhibitors of CA I and CA II [139] (Table 2).

### 1.5. 3,3′-diindolylmethane (DIM)

3,3′-Diindolylmethane (DIM, C17H14N2) is a known phytochemical compound derived from indole-3-carbinol (I3C) [140]. It is found in cruciferous vegetables like broccoli, cabbage, and kale [141]. DIM has an impact on signaling pathways and can regulate cell division, apoptosis, and angiogenesis in cancer cells [142]. It has been demonstrated that DIM inhibits PCGEM1 expression and induces apoptosis in prostate cancer [103]. Moreover, it has been observed that DIM indirectly suppresses the Akt/FOXM1 signaling cascade by regulating FOXM1 gene expression [143]. FOXM1 regulates various lncRNAs in some carcinomas [144]. Bioresponse formulated 3,3′-diindolylmethane (BR-DIM) decreases androgen receptor (AR) variants and AR3 expression in prostate cancer [103]. A study revealed that the combination of indolin-based compounds with sulfonamides can inhibit CA I, II, IV, and VII [145] (Table 2).

### 1.6. Epigallocatechin-3-galate (EGCG)

Epigallocatechin (EGCG, C15H14O7) is a known polyphenol flavonoid derived from almond and green tea [146,147,148,149,150,151]. This compound regulates the expression of non-coding RNAs in tumors and has notable anticancer, anti-inflammatory, and antioxidant features [38]. EGCG modulates various signaling pathways, such as NF-kB, MAPK, Akt, PI3K, PTEN, and mTORC1, as well as the expression of the estrogen receptor (ER) [152,153,154]. It has been shown that EGCG suppresses a lncRNA, AT102202, which downregulates the expression of 3-hydroxy-3-methylglutaryl coenzyme A reductase (HMGCR) in human hepatocytes, leading to the uptake of cholesterol by the liver [70]. A study showed that polyphenol Epigallocatechin upregulates CA IX in breast cancer cells, which may possess strong antioxidative and antiapoptotic properties [155]. It has also been demonstrated that EGCG as a content of flavonoids in green tea has a suppression effect on CA II [156] (Table 2).

### 1.7. Genistein

Genistein (C15H10O5), a dietary soy isoflavone, is another phytochemical compound with in vitro and in vivo antitumor effects [157]. It has shown some anti-proliferation effects on many types of human cancers, such as breast, renal, and prostate cancers [38,66,82,158,159,160]. Genistein modulates the expression level of HOTAIR in breast cancer, which consequently modulates the activity of the PI3K/Akt signaling pathway [161]. Genistein suppresses the progression of renal cancer by inhibiting HOTAIR [66]. It was found that the miR-141 expression was upregulated, while the HOTAIR expression was downregulated, by genistein in cancer cells [157]. In prostate cancer, genistein reduced the HOTAIR and miR-34a expression synergically. Another study also suggested that genistein has antitumor effects in colorectal cancer by affecting HOTAIR [162]. In addition, genistein induces apoptosis in cancer cells, including breast, prostate, gastric, lung, pancreatic, melanoma, and renal cancers, by inhibiting several signaling pathways, such as Wnt and Akt [82,163]. The decreased expression of HOTAIR leads to apoptosis, which has been induced by genistein in multiple types of cancer [88]. In this case, most studies considered the correlation between phyto-isoflavones and -oestrogens in cervix, ovariectomy, uterus, and liver cancers through the modulatory effect of genistein on CA II expression [164,165,166] (Table 2).

### 1.8. Quercetin

Quercetin (C15H10O7) is a polyphenolic flavonoid with chemopreventive properties. This dietary antioxidant is derived from several plants and fruits, such as red grapes, broccoli, and some berries. Quercetin downregulated the expression of DBH-AS1 in hepatocellular carcinoma through its antiproliferative and antioxidant activities [71,72,73]. It was reported that quercetin acts as an inhibitor in different signaling pathways like Akt/mTOR/P70S6K and PI3K/AkT [97,167,168]. Most studies have confirmed the inhibition activity of quercetin on CA isoforms, including CA I, II, III, IV, XII, and XIV [169,170,171]. Recently, quercetin-modified metal–organic frameworks (Zr-MOF-QU) as the novel type of Zr-MOF nanoparticles have shown excellent efficiency for CA IX inhibition in tumor cells [172] (Table 2). Zr-MOF-QU seems to be used successfully in radiotherapy.

### 1.9. Resveratrol

Resveratrol (3,4′,5 tri-hydroxystilbene) (C14H12O3) is a natural polyphenol compound found in various plants and herbs, including blueberries, raspberries, mulberries, and the skin of grapes [173]. Resveratrol has anti-inflammatory and antiproliferative properties, as well as antitumor effects on various human cancers [93,174], including prostate [113,175], thyroid [176], colorectal [177,178], breast [179,180], lung [181,182], and bladder cancers [93,95,111,113,183]. Resveratrol inhibits the AR signaling pathway in prostate cancer by affecting PCGEM1 and PRNCR1 [107,108,109,110]. Another prostate cancer study revealed that resveratrol is a reverse potent stimulator in the reduction of PCAT29 expression induced by a cancer cell line [175]. Synergistic growth inhibition activity of resveratrol and AK001796 has been demonstrated in lung cancer [14]. In another study, it was reported that resveratrol modulates the Wnt/β-Catenin signaling pathway by the downregulation of MALAT1 in colorectal cancer [95]. It has been demonstrated that the anticancer effect of resveratrol on estrogen receptor-α in breast cancer is due to the suppression of a lncRNA, u-Eleanor [111]. The aromatase inhibition property of resveratrol makes it a potential antitumor candidate in breast cancer treatment through the suppression of an oncogene, LINC00978 [92,93]. In the same study, LINC00978 functioned as a mediator for resveratrol to suppress the proliferation of breast cancer cells [93]. There is also evidence that the treatment of lung cancer with resveratrol results in the downregulation of AK001796 expression. Studies have revealed that polyphenol resveratrol could inhibit CA I‒XV in cancers, so CA II was inhibited more efficiently [135,184] (Table 2).

### 1.10. The Mechanisms of lncRNA Regulation by Phytochemicals

In recent years, several lncRNAs with interfering properties have been identified in different types of cancers. Thus far, the exact mechanism of lncRNA regulation in normal physiology or cancer cells is still unknown [185,186]. There is some evidence suggesting that lncRNAs are involved in the regulation of gene expression via transcriptional and post-transcriptional mechanisms and chromatin modification [9]. Furthermore, previous studies have defined that phytochemicals change the dysregulation of lncRNAs in various cancer types [187,188].

### 1.11. Transcriptional and Post-Transcriptional Regulation of lncRNAs

Experimental studies have revealed that there are several transcriptional factors regulating the expression of lncRNAs, and subsequently modulating pathological conditions in cancer. The studies have indicated that phytochemicals can adjust lncRNA expression via transcriptional regulation through various mechanisms. It has been shown that camptothecin decreases the transcription level of the HIF-1α gene in renal cancer [77]. Hypoxia inducible factor-1α is an important cell response modulator, which is regulated by lncRNAs and miRNAs [189,190].

TOP2A is a necessary element for the transcriptional activity of RNA polymerase II, which leads to a reduction of LS Pol II-mediated H19 transcription. Kujundzic and coworkers demonstrated that curcumin downregulates TOP2A expression and consequently inhibits H19 expression in tumor cell lines [191]. In another study, it was shown that curcumin regulates H19 through affecting the PI3K/Akt signaling pathway [192,193,194]. It was also shown that 3,3′-diindolylmethane inhibited the expression of PCGEM1 by banning its interaction with a nuclear RNA-binding protein, p54/nrb [103]. EGCG suppresses the promoter of the Cu(I) transport gene 1 (CTR1) in cancer cells, while it induces it through NEAT1, which is associated with hsa-miR-98-5p [195,196,197,198]. Furthermore, HOTAIR upregulates c-Myc in breast and ovarian cancers, which in turn promotes cancer cell proliferation [7]. Genistein downregulates the expression of HOTAIR at the transcription level in several cancers. The AR activation is a significant element in castration-resistant prostate cancer (CRPC) and increasing the expression level of HOTAIR [199].

### 1.12. Chromatin Modification by lncRNAs

lncRNAs are vital regulators of the genome structure, are able to interact with chromatin-modifying enzymes, and control the chromatin structure and accessibility to genetic information through reprogramming mechanisms [200,201]. The DNA methylation of genes inhibits the regulation of histone-modifying enzymes, which contributes to prostate cancer progression [175]. Several lncRNAs, such as PTENP1, Linc00963, PCGIM1, PRNCR1, CBR-3AS1, CTP1AS, GAS5, ANRIL, ANRASSF1, and PCAT1, upregulate the proliferation of cancer cells [59,74,102,175,202,203,204,205,206,207,208,209,210]. Resveratrol blocked the reduction of PCAT29 expression of this lncRNA in hepatocellular carcinoma [98]. HOTAIR can act as a mediator of proliferation, migration, invasion, and apoptosis in breast, liver, and colon cancer metastasis through genetic regulation [62,86,211]. Experimental studies have shown that curcumin can repress metastasis and invasion via epigenetic modulation [212]. Generally, lncRNAs are impartible vital molecules that are involved in gene modification and reprogramming. Phytochemicals, with their regulatory effects on lncRNAs, can be helpful as natural drugs for various cancer therapies.

## 2. Discussion

The idea of chemoprevention instead of chemotherapy was suggested by Moon et al. in 1979 for the prevention of breast cancer in rats using *N-*(4-Hydroxyphenyl)retinamide as a new retinoid compound [213]. Several studies proved this opinion sound during the subsequent decades, and studies were focused on discovering effective molecular targets for the modulatory function of phytochemicals.

lncRNAs are newly discovered regulators of cell functions, which have attracted considerable attention in biological sciences. Recently, researchers have discovered numerous lncRNAs in humans and animals, while their precise function is still unknown. At the moment, we are able to detect and analyze some regulatory functions of lncRNAs in cells. They are clearly crucial modulators of cell proliferation in cancer, and thus research on these molecules may open new avenues for cancer therapy. Previous studies have shown that lncRNA-low expression in tumors (lncRNA-LET) is involved in the inhibition of cell proliferation and cancer and metastasis suppression [99,100,214], while an opposite correlation was found between CA IX (an endogenous hypoxia marker and metabolism reprogramming factor) and lncRNA-LET during hypoxia in hepatocellular carcinoma [101,215]. Hypoxia induces cellular responses during cancer progression, including the overexpression of CA IX due to HIF-1α and HIF-1β stabilization through the protein kinase A (PKA) signaling pathway [216,217]. On the other hand, lncRNAs can induce the overexpression of CA XII through the PKA signaling pathway in fibrolamellar carcinoma [217]. In addition to these discoveries in cancer, an association has been found between CA II in the network of regulators and 16 lncRNAs in thoracic aortic dissection [218].

Phytochemicals have exhibited properties to change the level of lncRNAs involved in cancer induction and progression by regulating lncRNA expression through different signaling pathway mechanisms. These herbal drugs repress the proliferation and survival of cancer cells. Importantly, they are impressive natural compounds with no signs of toxicity or side effects. They can be applied simultaneously with some chemotherapy compounds in cancers, which may significantly improve the overall therapy outcome. The medicinal properties of phytochemicals have been shown not only in cancers, but also in a variety of chronic diseases, such as Alzheimer’s disease, cardiovascular diseases, diabetes, ocular diseases, and rheumatoid arthritis, in which they are capable of curing the dysregulation of lncRNAs [97,219,220].

Whithin this context, another recommended cancer therapeutic approach is called Acridine Orange-photodynamic therapy (AO-PDT), which was exploited by Kusuzaki et al. in 2017 [221]. In this cancer therapy method, AO was delivered by natural nanovesicles (exosomes) and released by macrophages, which consequently increased the uptake of AO by cancer cells. For the implementation of PDT in CA inhibition, a sulfonamide derivative of AO was delivered to tumor cells for the inhibition of cancer-related CA IX and XII [222,223,224].

One of the most important reasons for cancer relapse is the extracellular acidity of the tumor’s microenvironment, which can strongly influence cancer progression [21,225,226]. On the other hand, acidosis is a critical factor in the progression of tumors through promoting metastasis. One of the therapeutic strategies agaisnt tumor acidity is the inhibition or targeting of tumor acidity by diet buffers, sequestering agents in an acidic microenvironment, and developing proton pump inhibitors (PPIs). The acidic microenvironments are prepared by proton transporters, including V-ATPase, the Na+/H+ exchanger (NHE), monocarboxylate transporters (MCTs), and CA IX [227,228]. Since pKa for CA IX and XII was ˂6.5 and 7.1, respectively, CA IX is more active at a low pH. Hence, the inhibition of CA IX by low toxicity-inhibitors, including synthetic compounds and phytochemicals, or applying a combinational therapy method and their successful delivery to tumor acidic microenvironments by exosome nanovesicles is a therapeutic approach in cancers to overcome microenvironmental acidification [222]. This method has shown significant promise, which can accelerate the development of novel cancer treatment options.

On the other hand, there are some concerns about phytochemicals, like their poor bioavailability and limited efficiency, so novel formulations of these herbal drugs, such as combinations with adjuvants, liposomes, and nanoparticles, are needed to improve the efficacy of the phytochemicals for the modulation of lncRNAs in cancer. Although the phytochemicals have modulatory effects on lncRNAs, and could thus be useful in cancer prevention, the other biological effects of these compounds on other targets are inevitable. In parallel to the effects of phytochemicals on lncRNAs, the cancer-related CAs can also be inhibited efficiently. It reveals that some unprecedented targets are affected by phytochemicals within cancer prevention and/or treatment. On the other hand, it is obvious that cancer therapy with phytochemicals is a new area of science and research about its mechanisms of effect is still at a developing stage. More studies are needed to understand the relationship between the mentioned herbal drugs and their effects in normal and tumor cells. It is clear that more studies, including in vitro and in vivo tests, are needed to shed some new light on this research path.

## Figures and Tables

**Table 1:** Studied long non-coding RNAs (lncRNAs) in different types of cancer.

lncRNA	Cancer	Ref
**AF086415**	Nasopharyngeal carcinoma	[68,69]
**AK095147**	Nasopharyngeal carcinoma	[68,69]
**AK001796**	Thyroid cancer, Lung cancer	[14]
**AK056098**	Nasopharyngeal carcinoma	[68,69]
**AK294004**	Nasopharyngeal carcinoma	[68,69]
**AT102202**	Liver cancer	[70]
**DBH-AS1**	Hepatocellular carcinoma	[71,72,73]
**GAS5**	Gallbladder carcinoma, Breast cancer, Prostate cancer	[2,74]
**HULC**	Liver cancer	[75,76]
**HIF-1α**	Renal cancer	[77,78]
**HOTAIR**	Ovarian cancer, Renal cancer, Pancreatic cancer, Prostate cancer, Hepatocellular carcinoma, Nasopharyngeal carcinoma, Breast cancer, Lung cancer, Thyroid cancer, Gallbladder cancer	[5,11,13,65,67,79,80,81,82,83,84,85,86,87,88]
**H19**	Colorectal cancer, Pancreatic cancer	[89,90,91]
**LINC00978**	Lung cancer	[14,92,93]
**MALAT1**	Oral cancer, Bladder cancer, Colorectal cancer, Osteosarcoma	[94,95,96,97]
**MEG3**	Hepatocellular carcinoma	[98]
**RNA-LET**	Nasopharyngeal carcinoma	[99,100,101]
**PCGEM1**	Prostate cancer	[102,103]
**PVT1**	Pancreatic cancer	[104,105,106]
**PRNCR1**	Prostate cancer	[107,108,109,110]
**RP1-179N16.3**	Nasopharyngeal carcinoma	[68,69]
**u-ELEANOR**	Breast cancer	[111]

**Table 2:** Long non-coding RNAs (lncRNAs) and carbonic anhydrases (CAs) affected by phytochemicals.

Phytochemicals	lncRNAs	Carbonic Anhydrases (CAs)	Ref
Camptothecin (CPT)	HIF-1α	CA IX	[118,119,120,121]
Curcumin	GAS5, HOTAIR, H19, AF086415, AK095147, RP1-179N16.3, MUDENG, AK056098, AK294004	CA II, CA IX, CA XII	[134,135,136,137,138,139]
3,3′-diindolylmethane (DIM)	PCGEM1, FOXM1	CA I, II, IV, VII	[145]
Epigallocatechin-3-galate (ECGC)	AT102202	CA II, IX	[155,156]
Genistein	HOTAIR	CA II	[164,165,166]
Quercetin	DBH-AS1	CA I, II, III, IV, XII, XIV	[169,170,171,172]
Resveratrol	PCGEM1, PRNCR1, PCAT29, AK001796, MALAT1, u-Eleanor, LINC00978	CA I‒XV	[135,184]
